# The frequency of UTIs in people who undertake intermittent catheterization: A systematic review: Floriana D'Ambrosio

**DOI:** 10.1093/eurpub/ckac130.217

**Published:** 2022-10-25

**Authors:** F D'Ambrosio, F Orsini, A Scardigno, C Pappalardo, R Ricciardi, GE Calabrò

**Affiliations:** 1 Section of Hygiene, University Department of Public Health, Università Cattolica del Sacro Cuore, Rome, Italy; 2 Graduate School of Health Economics and Management, Università Cattolica del Sacro Cuore, Rome, Italy; 3 VIHTALI, Spin-Off of Università Cattolica del Sacro Cuore, Rome, Italy

## Abstract

**Background:**

Intermittent Catheterization (IC) is a common procedure used for the management of incomplete bladder emptying in various diseases such as spinal cord injury, multiple sclerosis and benign prostatic hypertrophy. Catheterization is associated with several complications and particularly with an increased risk of developing urinary tract infections (UTIs) responsible for high morbidity worldwide and significant costs to health systems and society.Today, this health problem is still underestimated. Therefore, the aim of this study was to summarize the available evidence on the clinical and epidemiological burden of UTIs among patients performing IC.

**Methods:**

A systematic literature review was performed querying two online database (PubMed,Web of Science) from January 2012 to January 2022. All studies in English language and focused on the clinical-epidemiological burden of UTIs related to IC in the adult population were included.

**Results:**

Overall, 43 studies were considered. It was described a range of UTIs from 26% to 63%, with an increased number of hospital admissions and length of stay. UTIs were more common in patients with spinal cord injuries (about 40%) and with multiple sclerosis (24-34%).The main risk factors associated with UTIs were catheter reuse, type of catheter and catheterization procedure adopted.

**Conclusions:**

Data on IC-associated UTIs are still limited. Estimating the UTIs load in patients with IC could support healthcare professionals to identify the most appropriate type of catheter to reduce the risk of this important complication. Proper management of catheterization could improve patients’ quality of life and also reduce the impact of diseases associated with this procedure on health systems and society.

**Key messages:**

- Catheterization is associated with an increased risk of UTIs causing morbidity and relevant costs to health systems and society.
- Knowing the burden of IC-associated UTIs could support better patients management in terms of suitable procedures to apply and more appropriate catheters to use.

